# A Polysaccharide from Dried Tangerine Peel: Structural Characterization and Alleviation of Gastric Injury by Modulating Oxidative Stress and Inflammatory Responses

**DOI:** 10.3390/foods15111837

**Published:** 2026-05-22

**Authors:** Huihui Li, Hao Wu, Yixia Chen, Yinyin Feng, Xiaoyang He, Huiqing Sun, Meng Meng

**Affiliations:** State Key Laboratory of Food Nutrition and Safety, Key Laboratory of Food Nutrition and Safety, Ministry of Education, College of Food Science and Engineering, Tianjin University of Science and Technology, No. 29, 13th Avenue, Tianjin Economy Technological Development Area, Tianjin 300457, China; lhh010309@163.com (H.L.); 18713696082@163.com (H.W.); chenyixiia@mail.tust.edu.cn (Y.C.); fengyin-yin2022@163.com (Y.F.); bunny_he@126.com (X.H.)

**Keywords:** dried tangerine peel, polysaccharide, structure, alleviation of gastric injury

## Abstract

Polysaccharides are important bioactive components of dried tangerine peel, exhibiting antioxidant, anti-inflammatory, and hypoglycemic activities. However, the ability of dried tangerine peel polysaccharides to alleviate gastric injury remains insufficiently understood. Therefore, the structure and alleviation of gastric injury induced by dried tangerine peel polysaccharides were explored in this study. Firstly, DTPP-4 was purified from dried tangerine peel. As shown in the HPLC chromatogram, DTPP-4 is a homogeneous polysaccharide with a mean molecular weight of 1.35 × 10^3^ kDa. DTPP-4 was mainly composed of L-Rha, L-Ara, D-Gal, and D-Gal*p*A with percentages of 10.56%, 9.15%, 4.83%, and 75.45%, respectively. Methylation and NMR results suggested that DTPP-4 was a pectic polysaccharide with →4)-α-D-Gal*p*A-6-OMe-(1→ and →4)-α-D-Gal*p*A-(1→ as the backbone. The alleviation of gastric injury of dried tangerine peel polysaccharide was evaluated in ethanol-induced acute gastric injury mice. Based on the macroscopic images of gastric tissues and the area of gastric tissue injury in mice, the dried tangerine peel polysaccharide can reduce the mouse gastric lesion area and alleviate gastric tissue pathological damage. Histopathological analysis of H&E and PAS staining revealed that the dried tangerine peel polysaccharide could ameliorate the disordered arrangement and necrosis of epithelial cells, reduce inflammatory cell infiltration, and thus alleviate gastric injury. Dried tangerine peel polysaccharide confers gastroprotection by modulating MPO and PGE2 levels, reducing MDA accumulation, enhancing SOD and CAT antioxidant activities, suppressing IL-1β and TNF-α secretion, and upregulating IL-10 expression. These findings provide a theoretical foundation for subsequent structure–activity relationship investigations and provide empirical support for the subsequent development and practical application of this polysaccharide.

## 1. Introduction

Gastric injury falls into acute and chronic categories [1]. Mucosal hyperemia, edema, erosion, and hemorrhage serve as typical features of acute gastric injury, and they are often triggered by excessive or episodic alcohol intake [2]. Gastric injury can substantially impair patients’ physical and psychological well-being and may impose a considerable healthcare burden. At present, multiple drugs, including H_2_-receptor antagonists, proton-pump inhibitors (PPIs), M_1_ receptor antagonists, and mucosal protectants, are extensively adopted in the treatment of gastric ulcers [3]. However, these drugs only provide symptomatic relief and frequently cause adverse reactions following long-term administration [4,5,6]. Consequently, considerable research interest has focused on identifying efficacious and safe natural agents for the prevention and mitigation of gastric injury. Numerous extracts obtained from edible and medicinal plants have been demonstrated to safeguard gastric mucosa and attenuate experimental gastric lesions [7,8,9,10]. As prevalent natural constituents ubiquitously existing in plants, polysaccharides exhibit diverse bioactivities, such as antioxidation, anti-inflammation, and immunomodulatory activity [11,12,13,14]. Citrus polysaccharides, particularly pectic-Gal*p*A-rich polymers, possess strong antioxidant and anti-inflammatory activities [15], which represent key mechanisms underlying the protection of gastric mucosa against ethanol-induced oxidative injury and inflammation [16]. These studies suggest that citrus polysaccharides may provide multi-target protection of the gastric mucosa [17].

In practical production, mature fruits of *Citrus reticulata* and its cultivars are collected, with peels stripped and air-dried to obtain dried tangerine peel [18]. The abundance of bioactive constituents, including flavonoids, limonoids, volatile oils, and pectin, endows dried tangerine peel with considerable potential for application in the food and pharmaceutical sectors. These functional components account for its pronounced antioxidant, anti-inflammatory, and antiproliferative bioactivities [19].

As one of the pivotal functional components in dried tangerine peel, polysaccharides exhibit excellent biocompatibility, low toxicity, and structural diversity [20]. Accumulating studies have demonstrated that dried tangerine peel polysaccharides possess prominent potential in free radical scavenging, immunomodulation, inflammatory response alleviation, and intestinal microenvironment improvement [21,22,23]. For instance, Lin et al. [24] demonstrated that DTPP promotes wound repair in zebrafish and mice through macrophage recruitment. In recent years, existing research on dried tangerine peel polysaccharides has mainly focused on extraction technologies, isolation and purification strategies, and biological activities evaluated via in vitro assays and animal models [21]. Although the chemical characteristics and partial biological functions of dried tangerine peel polysaccharides have been systematically explored, investigations regarding their regulatory roles in gastric mucosal barrier function, antioxidant defense, and inflammatory response mechanisms remain insufficient.

Therefore, the structure and alleviation of gastric injury induced by dried tangerine peel polysaccharides were explored in this study. Firstly, DTPP-4 was purified from dried tangerine peel. The structural characteristics of DTPP-4 were further elucidated by FT-IR, IC, methylation analysis, and NMR. Furthermore, an ethanol-triggered acute gastric injury mouse model was built to assess the gastroprotective effect of dried tangerine peel polysaccharides. The evaluation systems included macroscopic lesion observation, histopathological examination (H&E and PAS staining), and the detection of inflammation-related biomarkers and oxidative stress indices, involving MDA, SOD, CAT, TNF-α, IL-1β, IL-10, MPO, and PGE_2_. This work provides structural information on a high-molecular-weight pectic polysaccharide from dried tangerine peel, and offers in vivo evidence that this polysaccharide alleviates ethanol-induced gastric injury through coordinated regulation of oxidative stress and inflammatory responses. The manuscript provides a theoretical foundation for exploring its structure–activity relationship and supplies experimental evidence for subsequent development and practical utilization.

## 2. Materials and Methods

### 2.1. Materials

Dried tangerine peel was purchased from Jiangxi Furei Traditional Chinese Medicine Slices Co. Ltd. The PAS staining kit for glycogen was obtained from China Beijing Land Bridge Technology Co. Ltd. Paraffin was purchased from Leica Microsystems (Shang-hai) Trading Co. Ltd. Carbazole was obtained from China Shanghai Titan Scientific Co. Ltd. DEAE cellulose, Sephadex G-100 column, polysaccharide standards, monosaccha-ride standards and ELISA kits were purchased from China Beijing Solarbio Science & Technology Co. Ltd.

### 2.2. Preparation and Purification of Dried Tangerine Peel Polysaccharide

#### 2.2.1. Preparation of Dried Tangerine Peel Polysaccharide

Polysaccharides were extracted from dried tangerine peel by hot-water extraction [19]. 10.00 g samples with distilled water (1:20, *w*/*v*) were incubated in an 80 °C water bath for 2 h, then centrifuged at 3000 rpm for 10 min, and the supernatant was collected. The residue was subjected to two additional extraction cycles under identical conditions. Supernatants were concentrated via rotary evaporation, and the mixture was then combined with anhydrous ethanol. The resulting precipitate was redissolved in distilled water, residual ethanol evaporated, and the solution was diluted to a fixed volume to prepare the crude polysaccharide liquid. The efficiency of hot-water extraction was calculated to be 17.78 ± 0.12%.

A crude polysaccharide solution was deproteinized by the Sevag method [25]. UV–Vis detection was performed at 200–400 nm.

#### 2.2.2. Purification of Polysaccharide from Dried Tangerine Peel

Crude polysaccharide solution (20 mg/mL) was loaded onto a DEAE-52 cellulose column. [26]. The column was eluted sequentially with NaCl solutions ranging from 0 to 0.4 mol/L, and fractions corresponding to the major elution peaks with the highest polysaccharide content were pooled. A 40 mg/mL polysaccharide sample was applied to the Sephadex G-100 column, while the purity of the primary fraction was confirmed through high-performance liquid chromatography (HPLC) equipped with a refractive index detector. The resultant pure polysaccharide was designated as DTPP-4.

Using a UV–Vis spectrophotometer, a comprehensive wavelength analysis on a DTPP-4 solution across the 200 to 400 nm spectrum.

The molecular weight of DTPP-4 was measured by HPLC [27]. Dextran standards of 10, 40, 70, 500, and 2000 kDa were used. Each standard and DTPP-4 were dissolved and analyzed by HPLC to obtain retention times. A standard curve was established with retention time (0–20 min) on the *x*-axis and logarithmic molecular weight on the *y*-axis. Additionally, the sulfuric acid–carbazole assay was applied to quantify its uronic acid content [28].

### 2.3. Structural Characterization of DTPP-4

#### 2.3.1. Fourier-Transform Infrared Spectroscopy (FT-IR) Analysis

The DTPP-4 sample was thoroughly ground with spectral-grade KBr powder and pressed into a slice. FT-IR spectra were recorded on an IS50 spectrometer (Nicolet, USA) across the range of 4000–400 cm^−1^ with 32 scans accumulated [29,30].

#### 2.3.2. Analysis of Monosaccharide Composition

Monosaccharide constituents were analyzed via ion chromatography (IC) on a Dionex™ CarboPac™ PA20 column [30]. DTPP-4 underwent sealed acid hydrolysis at 115 °C for 3 h under nitrogen. Excess TFA was removed through repeated methanol co-evaporation under nitrogen flow. And the dried hydrolysate was redissolved in ultrapure water, filtered, and analyzed by IC. Monosaccharides were identified and quantified by comparison with monosaccharide reference standards based on retention times and peak areas.

#### 2.3.3. Methylation Analysis

Methylation analysis of DTPP-4 was conducted following a previously reported method [31,32,33,34,35]. Carboxyl groups were reduced, and complete methylation was performed using NaH/DMSO and methyl iodide, monitored by FT-IR until O-H peaks disappeared. Subsequent acid hydrolysis, reduction, and acetylation were carried out sequentially. Target derivatives were extracted with dichloromethane and determined by GC-MS. Linkage types were deduced from the characteristic fragmentation patterns and retention times.

#### 2.3.4. NMR Analysis

DTPP-4 (50.00 mg) was dissolved in D_2_O (1.00 mL) and ^1^H-NMR, ^13^C-NMR, HSQC, and COSY spectra were subsequently acquired [36].

#### 2.3.5. Congo Red Analysis

UV–Vis spectra of DTPP-4 and Congo red solutions were separately detected at ambient temperature. Gradient NaOH solutions were then blended into the mixed system. After 10 min incubation, all samples were scanned from 400 to 600 nm via a dual-beam ultraviolet spectrophotometer. The spectral variation trends were used to confirm the helical conformation of DTPP-4 [37].

### 2.4. Animal Experimentation and Design

All animal experiments were approved by the Animal Ethics Committee of Tianjin University of Science and Technology (Approval No. 2024025). In total, 72 male Kunming mice aged 3–4 weeks with a body weight of 18–20 g were obtained from SPF Biotechnology Co., Ltd., Beijing (Production License: SCXK (Jing) 2024-0001). All mice were raised in SPF-grade breeding rooms under standardized conditions [38]. Following the blinding procedures, 72 mice were randomly divided into six groups: control group (Control), model group (Model), positive control group (Positive), low-dose group (DL), medium-dose group (DM), and high-dose group (DH).

Normal saline was intragastrically administered daily to the control and model groups. The positive group was gavaged with 20 mg/kg omeprazole solution. To investigate the effects of DTPP on experimental mice, three dosage groups were established: low, medium, and high doses of 100.00, 200.00, and 400.00 mg/(kg·d) were separately administered by gavage.

The intragastric administration was continued for 14 days. On the last day, apart from the control group, all mice were gavaged with 0.10 mL/10.00 g absolute ethanol to establish acute gastric injury models.

### 2.5. Area of Gastric Tissue Injury in Mice

Gastric tissues were measured and photographed for subsequent quantification of the gastric lesion area (mm^2^) using ImageJ software (version 1.54).

### 2.6. H&E Staining of Mouse Gastric Tissue

Gastric tissues were stored in 4% paraformaldehyde, dehydrated, and embedded in paraffin. The tissues were sectioned and stained with hematoxylin and eosin.

### 2.7. PAS Staining of Mouse Gastric Tissue

Gastric tissues were stored in 4% paraformaldehyde, followed by dehydration and paraffin embedding. The tissues were sectioned and stained with PAS reagent.

### 2.8. Measurement of Gastric Tissue-Related Biomarkers

The levels of myeloperoxidase (MPO) and prostaglandin E2 (PGE2) in tissue samples were detected using commercial ELISA kits. The entire detection process was strictly conducted in accordance.

### 2.9. Measurement of Oxidative Stress-Related Markers

Levels of Oxidative Stress-Related Markers (MDA, SOD, CAT) in gastric tissues were detected using commercial assay kits. The entire detection process was strictly conducted in accordance.

### 2.10. Measurement of Inflammatory Responses

Levels of Inflammatory Responses (IL-10, IL-1β, TNF-α) in gastric tissues were quantified via commercial ELISA kits in strict compliance with the manufacturer’s guidelines.

### 2.11. Data Analysis

Data processing and statistical analysis were performed using Origin 2024 and SPSS 27.0. Group comparisons were conducted by one-way ANOVA with Tukey’s post hoc test, and distinct lowercase letters indicate significant differences (*p* < 0.05).

## 3. Results

### 3.1. Purification and Compositional Analysis of DTPP-4

**Figure 1:**
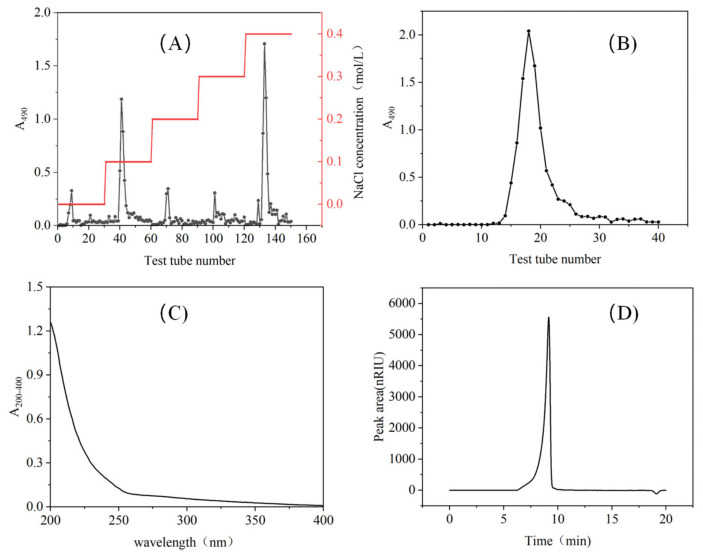
(**A**) Elution curve of crude polysaccharides via DEAE-52 column. (**B**) Elution curve of DTPP-4 on a Sephadex G–100 column. (**C**) UV–Vis spectrum of DTPP-4. (**D**) HPLC chromatogram of DTPP-4.

Figure 1A indicates that the fraction eluted with 0.4 mol/L NaCl displayed the highest peak intensity. The polysaccharide fractions eluted from the DEAE-52 ion-exchange column were desalted and lyophilized. And, after reconstitution to 40.00 mg/mL, the resulting solution was then loaded onto a Sephadex G–100 column. Its elution curve shows a single symmetric peak (Figure 1B), confirming that the obtained fraction was homogeneous and named DTPP-4.

The UV–Vis spectrum of DTPP-4 solution was recorded over 200–400 nm. As shown in Figure 1C, no absorption indicative of nucleic acids or proteins was detected.

As shown in Figure 1D, HPLC yielded a single symmetric peak, confirming the homogeneity of DTPP-4. The standard curve was established using retention time and logarithmic molecular weight for dextran markers. The equation of the calibration curve is: y = −3.248x + 29.061, with R^2^ = 0.9996. Substituting its retention time into the standard curve gave a molecular weight of 1.35 × 10^3^ kDa, and the calculated value was 85.81 ± 0.98%, indicating that DTPP-4 is an acidic polysaccharide.

### 3.2. Structural Characterization of DTPP-4

#### 3.2.1. FT-IR Analysis of DTPP-4

**Figure 2:**
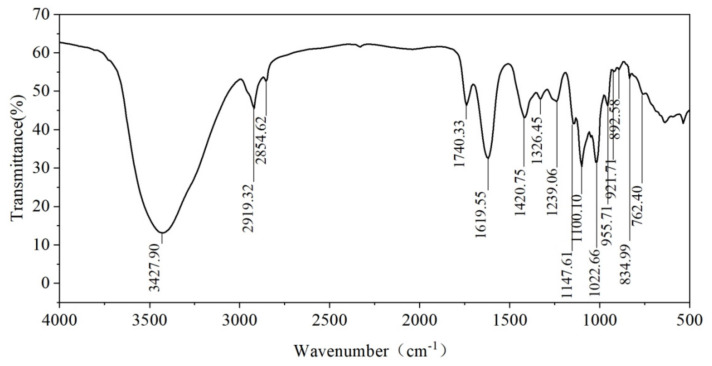
FT-IR spectrum of DTPP−4.

As illustrated in Figure 2, the absorption band at 3427.90 cm^−1^ was attributed to the O–H stretching vibration. Absorption bands at 2919.32 cm^−1^ and 2854.62 cm^−1^ were attributed to asymmetric C–H stretching, while the peak at 1740.33 cm^−1^ corresponded to C = O stretching vibration of carboxyl groups. verifying the existence of uronic acid, which was consistent with chemical analysis results.

#### 3.2.2. Monosaccharide Composition Analysis of DTPP-4

**Figure 3:**
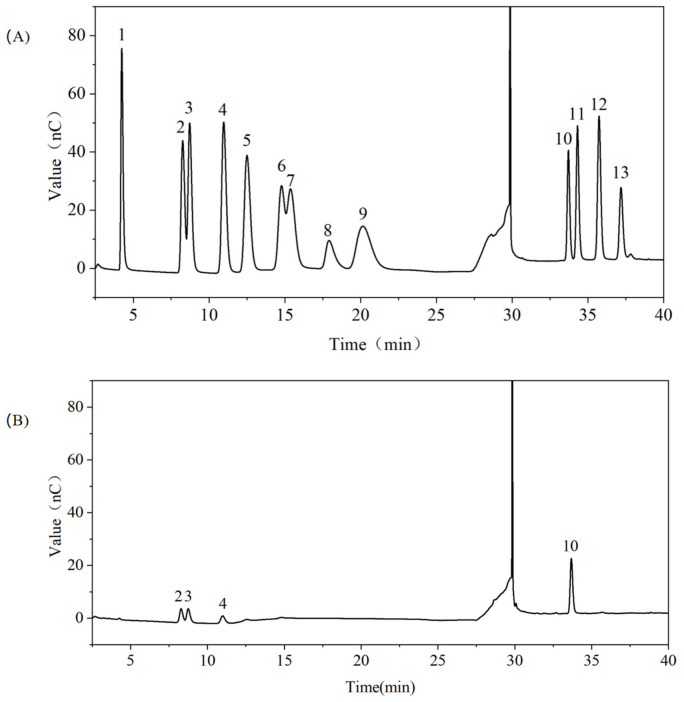
(**A**) DTPP-4 monosaccharide label and (**B**) DTPP-4 monosaccharide composition.

IC results (Figure 3) revealed the monosaccharide constituents of DTPP-4, including L-Rha, L-Ara, D-Gal, and D-Gal*p*A. The relative percentages of L-Rha, L-Ara, D-Gal, and D-Gal*p*A were 10.56%, 9.15%, 4.83%, and 75.45%, respectively. A high content of D-Gal*p*A (75.45%) demonstrated that DTPP-4 belongs to an acidic polysaccharide.

#### 3.2.3. Methylation Analysis of DTPP-4

**Figure 4:**
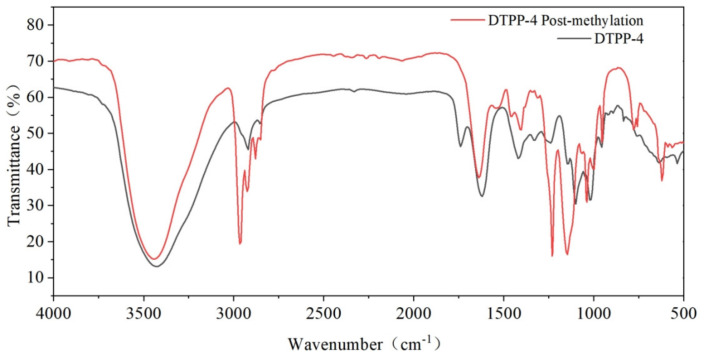
Infrared spectrum of DTPP−4 methylation.

Methylation analysis stands as a commonly employed core method in investigations of carbohydrate chain structures. After six cycles of methylation, FT-IR spectroscopy was used to confirm full methylation. Figure 4 indicates that the characteristic -OH around 3400 cm^−1^ obviously weakened, and a marked enhancement was observed in the intensity of C-H stretching vibrations occurring around 2900 cm^−1^, indicating that the methylation had achieved completeness. Thus, the material was deemed suitable for subsequent analysis and quantification. Subsequent GC-MS analysis identified four monosaccharide derivatives (Table 1): 2,3,6-O-Me_3_-galactitol, 6-deoxy-3,4-O-Me_2_-mannitol, 3,4,6-O-Me_3_-galactitol, and 2,5-O-Me_2_-arabinitol, with relative molar ratios of 6.4035: 1.0026: 0.4839: 0.9761. The results confirmed DTPP-4 contains four types of sugar residues: →4)-Gal*p*A-(1→, →2)-Rha*p*-(1→, →2)-Gal*p*-(1→, and →3)-Ara*f*-(1→, in which →4)-Gal*p*A-(1→ was the main chain. The results proved that DTPP-4 is a pectin-based polysaccharide.

#### 3.2.4. NMR Analysis of DTPP-4

**Figure 5:**
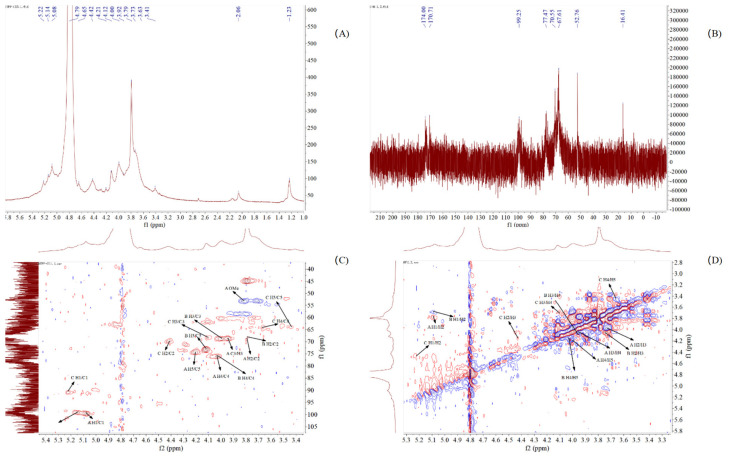
(**A**) ^1^H NMR, (**B**) ^13^C NMR, (**C**) COSY, and (**D**) HSQC spectra of DTPP−4.

The ^1^H, ^13^C NMR, COSY, and HSQC spectra of DTPP-4 are presented in Figure 5. The chemical shifts of each monosaccharide residue are listed in Table 2. Most hydrogen (H) signals in Figure 5A appeared at 3.01–5.9 ppm. which is characteristic of a polysaccharide structure. As shown in Figure 5D, the HSQC spectrum displays two strong and one relatively strong signal in the range of 90–110 ppm, specifically at 99.57/5.08 ppm, 99.25/5.14 ppm, and 90.85/5.22 ppm. These signals indicate that DTPP-4 primarily consists of three types of sugar residues, designated as residue A, residue B, and residue C based on their C1/H1 signals. This suggests that the glycosidic linkages in DTPP-4 are predominantly in the α-configuration [39]. Combined with methylation data and published references, residues A and B were identified as →4)-α-D-Gal*p*A-6-OMe-(1→ and →4)-α-D-Gal*p*A-(1→. Based on the C1/H1 signals, the H2–H5 signals can be identified in the COSY spectrum, and the C2–C5 signals can be located in the HSQC spectrum. For H6, which is typically not observed due to the lability of the carboxyl hydrogen and its propensity for substitution, no signal is expected. The signal at 5.22 ppm in the HSQC spectrum corresponds to the H1 of Rha*p*, while the signal around 17.0 ppm corresponds to the C6 of Rha*p*. Combined with the HSQC signal at 90.86/5.22 ppm and the ^13^C/^1^H signals at 16.41/1.23 ppm, the presence of a Rha structure in DTPP-4 is inferred. This linkage can be assigned as →2)-α-L-Rha*p*-(1→. Combined with C1/H1 signals, H2–H5 and C2–C5 signals were further identified via COSY and HSQC spectra. However, due to the low abundance of arabinose and galactose residues in DTPP-4 and the limited resolution of the NMR spectrometer, the signals for other positions of these residues could not be reliably assigned. In summary, NMR and methylation analyses revealed that DTPP-4 main chains consist of→4)-α-D-Gal*p*A-(1→ and →4)-α-D-Gal*p*A-6-OMe-(1→ residues, confirming its pectic nature.

**Table 2:** Chemical shift assignment of monosaccharide residues ^1^H and ^13^C NMR in DTPP-4.

Serial Number	Monosaccharide Residue	Chemical Shift (δ, ppm)						
		C1 H1	C2 H2	C3 H3	C4 H4	C5 H5	C6 H6	**OMe**
A	→4)-α-D-Gal*p*A-6-OMe-(1→	99.57 5.08	69.03 3.77	68.61 3.92	76.03 4.00	74.25 4.21	170.71 /	52.76 3.79
B	→4)-α-D-Gal*p*A-(1→	99.25 5.14	69.03 3.73	68.77 3.99	76.03 4.00	73.13 4.12	174.00 /	/
C	→2)-α-L-Rha*p*-(1→	90.86 5.22	69.85 4.22	68.54 4.10	64.11 3.63	63.99 3.41	16.41 1.23	/

Note: "/" indicates not detected.

#### 3.2.5. Congo Red Analysis of DTPP-4

**Figure 6:**
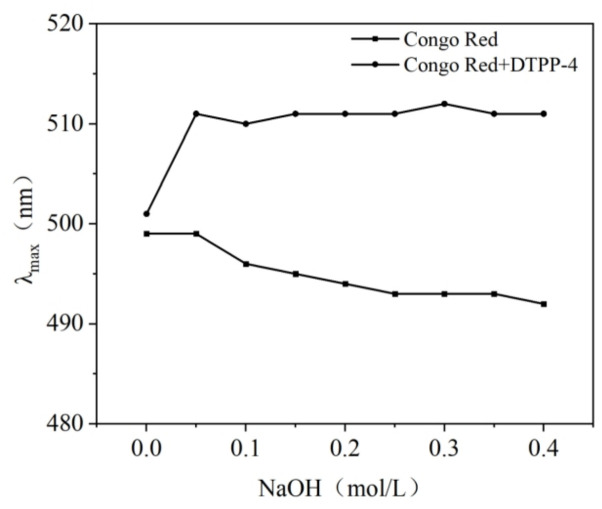
Wavelength changes.

Figure 6 indicates that the maximum absorption wavelength of Congo red mixed with DTPP-4 remained essentially unchanged relative to that of Congo red alone across the range of NaOH concentrations tested. Therefore, it is inferred that DTPP-4 possesses no helical structure under weakly alkaline conditions.

### 3.3. Alleviation of Gastric Injury of Polysaccharide from Dried Tangerine Peel

In this study, animal experiments were conducted to investigate the gastric injury-alleviating activity of dried tangerine peel polysaccharides. An alcoholic gastric injury model was established by inducing gastric injury in mice with ethanol. Methods including macroscopic observation, gastric injury area measurement, HE and PAS staining, detection of oxidative stress and inflammatory responses, as well as determination of gastric tissue-related factors, were employed to explore the effects of dried tangerine peel polysaccharides on gastric injury.

#### 3.3.1. Macroscopic Images of Gastric Tissues

**Figure 7:**
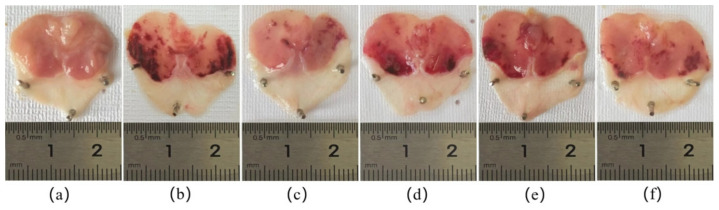
Macroscopic images of gastric tissue: (**a**) Control group; (**b**) Model group; (**c**) Positive group; (**d**) DL group; (**e**) DM group; (**f**) DH group.

As shown in Figure 7, severe gastric edema, hemorrhage, mucosal shedding, and extensive ulcers were observed in the model group. Such lesions were distinctly relieved in a dose-dependent manner in polysaccharide-treated and positive groups. These findings demonstrated that DTPP-4 effectively alleviated ethanol-triggered gastric damage in mice.

#### 3.3.2. Area of Gastric Tissue Injury in Mice

**Figure 8:**
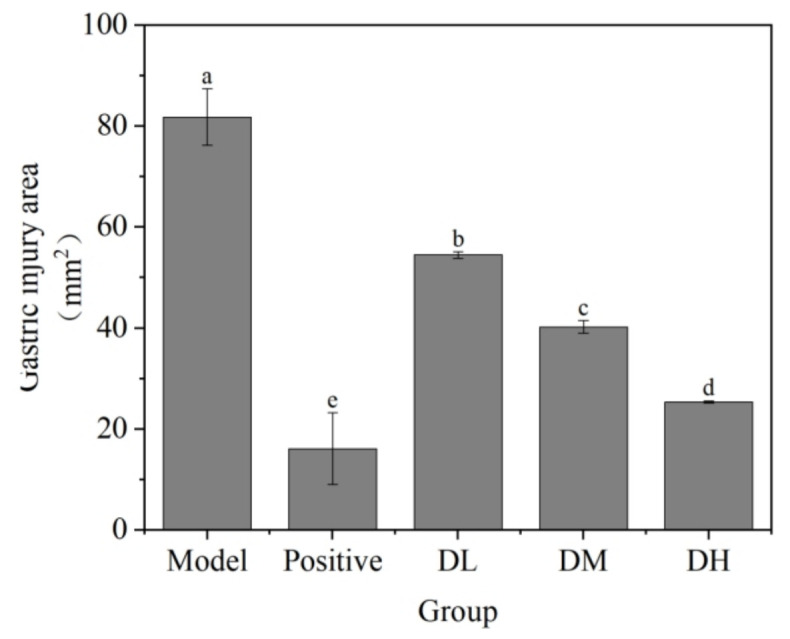
Area of gastric tissue injury. Note: the distinct lowercase letters indicate significant differences (*p* < 0.05).

As shown in Figure 8, gastric lesion areas were significantly reduced in both the polysaccharide-treated and positive control groups relative to the model group (*p* < 0.05), and exhibited a marked dose-dependent decrease. These results indicated that dried tangerine peel polysaccharides can effectively reduce gastric lesion area and alleviate gastric tissue damage in mice.

#### 3.3.3. H&E Staining Analysis

**Figure 9:**
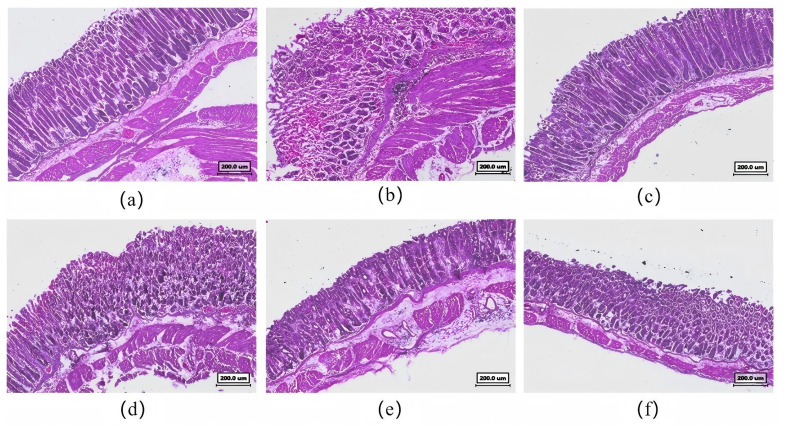
H&E staining of gastric tissue: (**a**) Control group; (**b**) Model group; (**c**) Positive group; (**d**) DL group; (**e**) DM group; (**f**) DH group.

Figure 9 indicated that the control group presented intact gastric mucosal structure with well-organized epithelium, submucosa, and serosa. No inflammatory infiltration or tissue edema was detected, and gastric glands were neatly arranged. In contrast, severe damage occurred to gastric mucosal epithelial cells in the model group, with extensive shedding and loss. The glandular structure was disrupted and disorganized. Compared with the model group, polysaccharide pretreatment markedly attenuated gastric tissue injury to varying degrees; the DH group exhibited the greatest alleviation of gastric damage.

#### 3.3.4. PAS Staining Analysis

**Figure 10:**
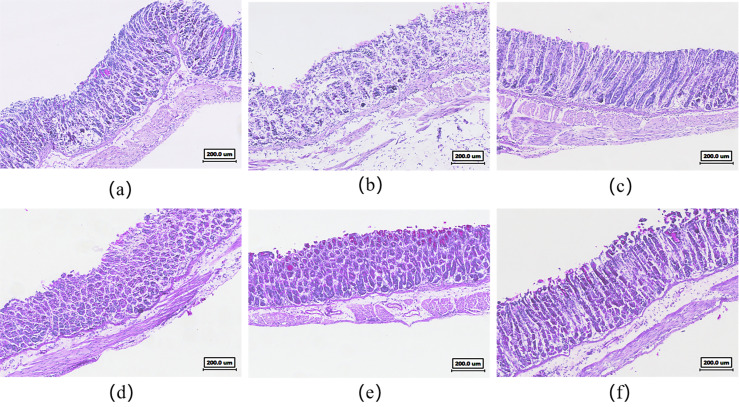
PAS staining of gastric tissue: (**a**) Control group; (**b**) Model group; (**c**) Positive group; (**d**) DL group; (**e**) DM group; (**f**) DH group.

Figure 10 indicates that intact and well-organized gastric glands were observed in the control group, with no signs of epithelial cell shedding or inflammatory cell infiltration. The Model group exhibited extensive epithelial cell sloughing, inflammatory cell infiltration, and vascular damage in the gastric mucosa, along with widened interstitial spaces and a disorganized overall arrangement of the gastric tissue. Relative to the model group, the DH group exhibited tightly arranged tissue cells and intact, orderly gastric glands, with only a small portion of the tissue lacking epithelial cells.

#### 3.3.5. Effects of DTPP on Gastric Tissue Biomarkers in Mice with Gastric Injury

**Figure 11:**
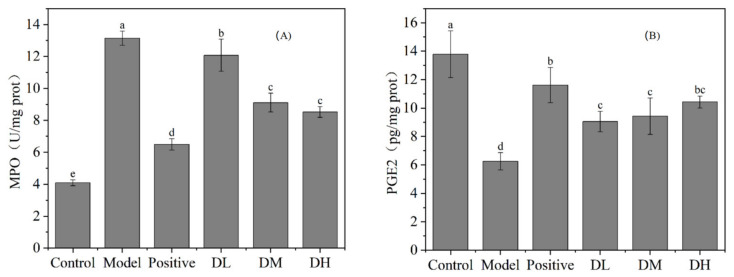
Changes in gastric tissue-related factors: (**A**) MPO; (**B**) PGE_2_ in mice. Note: the distinct lowercase letters indicate significant differences (*p* < 0.05).

Figure 11 reveals that MPO content was markedly elevated in the model group relative to the control group (*p* < 0.05). Polysaccharide pretreatment effectively suppressed MPO accumulation compared with model mice. Collectively, dried tangerine peel polysaccharides downregulated gastric MPO levels, thereby ameliorating ethanol-mediated gastric lesions.

The model group displayed distinctly reduced gastric PGE_2_ content (*p* < 0.05), suggesting impaired gastric microcirculation. By contrast, PGE_2_ levels were notably elevated in all polysaccharide intervention groups (*p* < 0.05). These data demonstrated that dried tangerine peel polysaccharides upregulated gastric PGE_2_ expression and further relieved ethanol-mediated gastric lesions.

#### 3.3.6. Effects of DTPP on Oxidative Stress-Related Markers in Gastric Injury Mice

**Figure 12:**
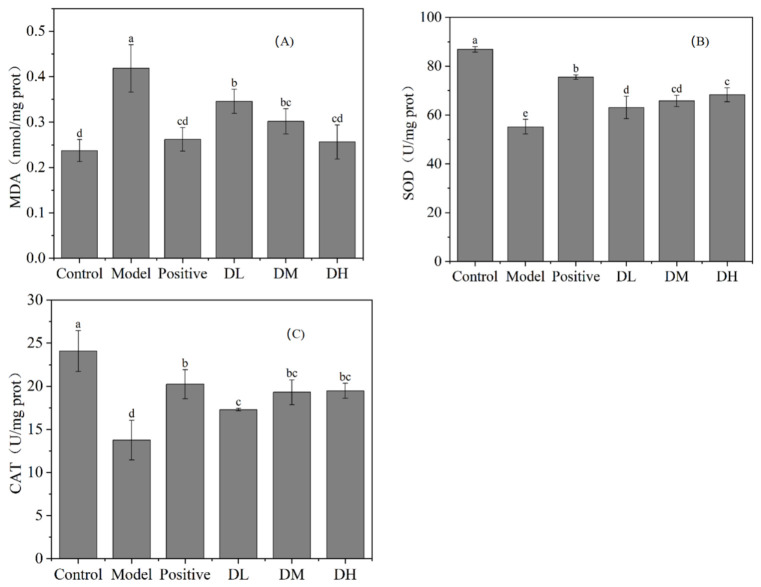
Changes in oxidative stress-related indicators: (**A**) MDA; (**B**) SOD; (**C**) CAT. Note: the distinct lowercase letters indicate significant differences (*p* < 0.05).

Figure 12 reveals that, compared with the model group, all intervention groups exhibited elevated CAT and SOD activities, as well as reduced MDA levels. These results revealed that dried tangerine peel polysaccharides regulated the oxidative stress markers (SOD, CAT, MDA), ultimately mitigating gastric damage.

#### 3.3.7. Effects of DTPP on Inflammatory Responses in Gastric Injury

**Figure 13:**
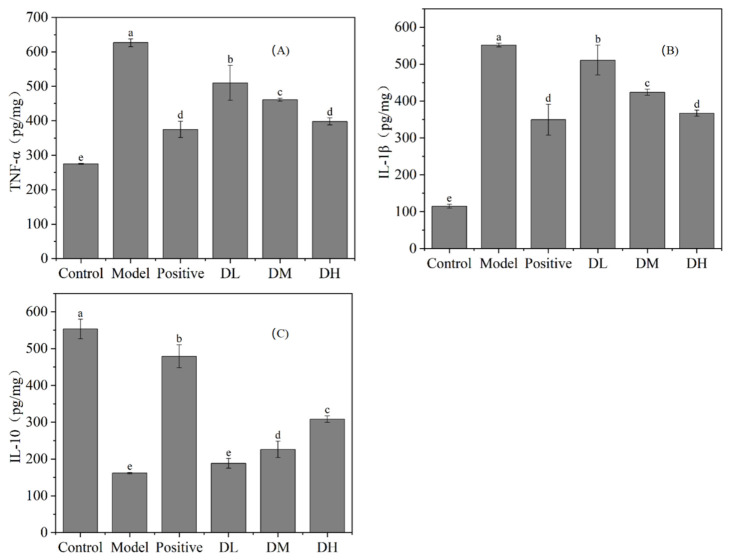
Changes in mouse inflammatory responses: (**A**) TNF-α; (**B**) IL-1 β; (**C**) IL-10. Note: the distinct lowercase letters indicate significant differences (*p* < 0.05).

Figure 13 showed that omeprazole and polysaccharide intervention effectively reversed the abnormal inflammatory alterations relative to the model group. The levels of the pro-inflammatory cytokines TNF-α and IL-1β were decreased in a dose-dependent manner. Meanwhile, gastric IL-10 levels were markedly upregulated after polysaccharide administration (*p* < 0.05). These results revealed that dried tangerine peel polysaccharides regulated inflammatory responses (TNF-α, IL-1β, IL-10), which further alleviated ethanol-induced gastric lesions.

## 4. Discussion

Although the chemical characteristics and partial biological functions of dried tangerine peel polysaccharides have been systematically explored, investigations regarding their regulatory roles in gastric mucosal barrier function, antioxidant defense, and inflammatory response mechanisms remain insufficient. Therefore, in this work, a homogeneous acidic pectin polysaccharide DTPP-4 was isolated and structurally identified from dried tangerine peel. DTPP-4 was rich in galacturonic acid and mainly contained →4)-α-D-GalpA-6-OMe-(1→ and →4)-α-D-GalpA-(1→ residues, indicating a typical pectic polysaccharide structure. In the ethanol-induced acute gastric injury model, dried tangerine peel polysaccharides significantly reduced gastric lesion area, ameliorated histopathological damage, enhanced antioxidant defense, and modulated inflammatory cytokine production. These findings suggest that dried tangerine peel polysaccharides have potential as a gastroprotective functional polysaccharide.

At present, studies on the structure of dried tangerine peel polysaccharides remain limited. Most existing studies mainly focus on fresh citrus peels, the extraction technology, while investigations on their fine structure are still scarce [24,40,41]. The molecular weight and structural characteristics of citrus polysaccharides reported in previous studies are different from those reported in the manuscript. Zhou et al. [42] adopted GC-MS to determine monosaccharide components. NMR results confirmed that polysaccharides from Citrus reticulata ‘Chachi’ peel were mainly composed of arabino galacturonan and side-chain-containing pectin, with multiple glycosidic linkages identified. Chen et al. [43] optimized extraction conditions to obtain purified peel pectic polysaccharide TPPs-2-1, and clarified its monosaccharide composition, molecular weight, and glycosidic bonds via FT-IR, IC, and HPGPC. Hence, a systematic structural characterization of purified DTPP is necessary, providing a theoretical foundation for exploring its structure–activity relationship and supplying experimental evidence for subsequent development and practical utilization.

Pectic polysaccharides with homogalacturonan and rhamnogalacturonan regions have been widely reported to display antioxidant and immunomodulatory activities [44,45]. Vogt et al. [46] studied lemon pectins with diverse methylation degrees (30%, 56%, 74%) to explore the effects of chain length and esterification level on Toll-like receptors and in vitro intestinal barrier function. Liu et al. [47] extracted and characterized Lycium ruthenicum polysaccharides (LRPs) via chemical assays and FT-IR, and evaluated their antioxidant activities in vitro. The high galacturonic acid content and predominant →4)-Gal*p*A-(1→ backbone of DTPP-4 conform to this typical structural feature. These findings suggest that this pectic structural feature may regulate oxidative stress markers (SOD, CAT, MDA), thereby attenuating ethanol-induced oxidative damage. Nevertheless, whether dried tangerine peel polysaccharides directly scavenge reactive oxygen species or act indirectly via cell signaling pathways remains to be further elucidated.

MPO is an enzyme distributed in neutrophils and monocytes. Increased MPO activity reflects inflammatory cell infiltration and the severity of the inflammatory response [38]. Accordingly, MPO activity can serve as an indicator for assessing inflammatory infiltration in gastric tissues. PGE_2_ serves a key role in safeguarding the gastric mucosa by facilitating mucosal repair [8]. Declined PGE_2_ levels are a typical characteristic of ethanol-induced gastric injury, and PGE_2_ content can effectively reflect the functional status of the gastric mucosal protective mechanism. Zhang et al. [48] identified a polysaccharide extract from Bletilla striata (BSP). The extracted BSP exhibited strong radical-scavenging abilities as assessed by 2, 2′-azino-bis (3-ethylbenzothiazoline-6-sulphonic acid) diammonium salt (ABTS) and Ferric Reducing Antioxidant Power (FRAP) assays. Moreover, BSP effectively inhibited gastric pro-inflammatory responses, as evidenced by the downregulation of tissue MPO activity. This polysaccharide also exerted prominent antioxidant effects, elevated the level of the gastric protective factor PGE2, and blocked the activation of the MAPK/NF-κB signaling pathway in gastric tissues. Consistent with the above findings, this study verified that dried tangerine peel polysaccharides could decrease gastric MPO accumulation and upregulate PGE2 levels, thereby ameliorating ethanol-triggered gastric mucosal damage in mice.

Gastric injury is strongly associated with oxidative stress responses [6]. As a hallmark metabolite of lipid peroxidation, MDA accumulation is considered a key indicator of ethanol-induced gastric lesions [47]. SOD and CAT are antioxidant enzymes within cells. In ethanol-induced gastric injury, ethanol triggers excessive free radical production, which suppresses SOD and CAT activities [25] and weakens the cellular free-radical-scavenging potential, consequently exacerbating oxidative injury in gastric tissues. SOD and CAT activities effectively reflect the antioxidant defense capacity of gastric tissues. Cai et al. [49] employed a D-galactose-induced aging mouse model for their investigation. Their findings demonstrated that tea flower polysaccharides (TFPSs) suppressed the overactivation of hippocampal microglia in aging mice. TFPS treatment significantly downregulated pro-inflammatory mediators (IL-6, TNF-α, IL-1β) and the nuclear transcription factor NF-κB. Concurrently, this polysaccharide enhanced antioxidant enzyme activities and reduced MDA accumulation. Evidence indicates that TFPS ameliorates gut microbiota dysbiosis, attenuates glial oxidative damage and neuroinflammation, and exerts anti-aging effects. Xia et al. [50] explored the protective efficacy and underlying mechanisms of Ulva prolifera polysaccharides (PUPs) against valproic acid (VPA)-triggered oxidative stress. In vitro assays validated that PUPs remarkably improved cell viability in VPA-injured models, strengthened cellular antioxidant capacity by elevating intracellular SOD and CAT activities, and concurrently reduced ROS and MDA levels. PUPs were therefore proven to possess prominent protective effects against VPA-mediated oxidative damage in neuronal HT22 cells.

Gastric injury is closely correlated with inflammatory processes, which participate in the initiation, progression, and recovery of gastric mucosal damage [32]. IL-1β and TNF-α are typical pro-inflammatory cytokines. They can trigger inflammatory cascades, promote inflammatory-cell infiltration, and stimulate the release of additional inflammatory mediators [33], thereby aggravating gastric mucosa inflammation. In addition, TNF-α can induce cell apoptosis and further impair gastric mucosal cells. The overexpression of IL-1β and TNF-α is a key indicator of ethanol-induced stomach damage, and their presence tends to correlate with the intensity of the local inflammatory reaction. By contrast, IL-10 serves as a key anti-inflammatory cytokine, which inhibits pro-inflammatory mediator release and relieves inflammatory tissue lesions. The level of IL-10 can effectively evaluate the anti-inflammatory capacity of gastric tissues. Bermudez-Brito et al. [51] found that chicory inulin and barley β-glucan downregulated IL-6 expression in type 2 (Th2) cells during co-culture with epithelial and dendritic cells (DCs), whereas chicory inulin inhibited IL-10 secretion, implying their potential pro-inflammatory tendency. As a component of plant polysaccharides, galacto-oligosaccharides could trigger regulatory T cells (Tregs) to secrete IL-10. Chatterjee et al. [52] verified the gastroprotective effect of water-soluble polysaccharides from Termitomyces eurhizus (TEps) against indomethacin-induced gastric ulcers in mice. Histological observations confirmed its ulcer-repairing ability. Such efficacy was attributed to elevated PGE_2_ synthesis via regulating COX-1 and COX-2, along with a shift from pro-inflammatory (TNF-α, IL-1β) to anti-inflammatory (IL-10) expression. Inflammatory responses are not merely simple inflammatory products but serve as key upstream activators of signaling pathways, which can initiate the activation of downstream pathways via specific membrane receptors. Numerous studies have demonstrated that polysaccharides can inhibit excessive inflammation and alleviate tissue damage by modulating the activation of inflammatory signaling pathways. Raish et al. [53] delved into the healing capacity and the ins and outs of how Momordica charantia polysaccharides (MCPs) combat ethanol-induced stomach sores in rodents. Pre-administration of MCP alleviated gastric lesions mainly through blocking NF-κB pathways. Zhang et al. [54] reported that Lyophyllum decastes polysaccharides (LDFPs) activated the Nrf2 pathway by upregulating Nrf2 and downregulating Keap1, and they further elevated HO-1 and CuZn-SOD expression. Meanwhile, LDFP inhibited TLR4 and NF-κB expression, thereby reducing IL-6 and TNF-α release and exerting hepatoprotective effects. Lin et al. [55] evaluated the therapeutic efficacy of polysaccharides from peach gum (PGP) in ulcerative colitis. The underlying molecular mechanisms were also elucidated. In vitro experiments showed that PGP regulated the secretion of inflammatory mediators in RAW264.7 cells. In vivo, PGP upregulated Ki67 expression to facilitate colonic epithelial cell proliferation and protected Caco-2 cell barrier function against LPS injury via mediating the PI3K/AKT pathway. It is therefore speculated in this study that dried tangerine peel polysaccharides may alleviate inflammatory responses by regulating levels of inflammatory cytokines and further modulating related signaling pathways.

From an application perspective, dried tangerine peel is already used as a food ingredient and traditional remedy, which facilitates the potential translation of its polysaccharide fraction into gastroprotective functional foods or nutraceuticals. For example, DTPP-4-enriched extracts could be incorporated into beverages, nutraceutical capsules, or meal-replacement products intended for populations at high risk of alcohol-related gastric discomfort. Future studies should also clarify the detailed branching patterns and conformational properties of DTPP-4 and delineate the signaling pathways through which it modulates oxidative stress and inflammation.

## 5. Conclusions

This research explored the architecture and gastroprotective effects of dried tangerine peel polysaccharides. A polysaccharide, designated DTPP-4, was obtained from dried tangerine peel. DTPP-4 is a homogeneous pectic polysaccharide possessing an average molecular mass of 1.35 × 10^3^ kDa, and its main chain is composed of →4)-α-D-Gal*p*A-(1→and →4)-α-D-Gal*p*A-6-OMe-(1→ glycosidic linkages. In addition, the gastroprotective capacity of dried tangerine peel polysaccharides was systematically assessed. The dried tangerine peel polysaccharide can alleviate gastric injury by modulating the levels of MPO and PGE_2_, decreasing MDA accumulation and improving SOD and CAT activities, decreasing IL-1β and TNF-α secretion while upregulating IL-10 levels. The manuscript provides structural information on a high-molecular-weight pectic polysaccharide from dried tangerine peel, and offers in vivo evidence that this polysaccharide mitigates gastric injury by coordinated regulation of inflammatory responses and oxidative stress. These findings provide structural and biological evidence supporting the potential application of dried tangerine peel polysaccharides as gastroprotective functional food ingredients. Future research can be further expanded by focusing on the structural properties of pectin polysaccharides, embedding technologies, and delivery systems. Future work can focus on refining the structure of DTPP-4. Elucidating the upstream signaling pathways and cellular targets involved in DTPP-4 antioxidant and anti-inflammatory actions. And relying on the inherent amphipathy, biocompatibility, and gelation properties of pectin, embedding technologies can be introduced to improve the stability of polysaccharides. Meanwhile, co-embedding and synergistic delivery systems of pectin polysaccharides with other bioactive components of dried tangerine peel can be explored to alleviate gastric injury.

## Figures and Tables

**Table 1:** Methylation analysis results of DTPP-4.

Retention Time (min)	Methylated Sugar	Type of Linkage	Mass Fragments (m/z)	Relative Molar Ratios
10.942	1,4,5-O-Ac_3_-2,3,6-O Me_3_-galactitol	→4)-Gal*p*A-(1→	42, 55, 69, 87, 99, 110, 125, 131, 173, 233	6.4035
7.426	1,2,5-O-Ac_3_-3,4-O- Me_2_-rhamnitol	→2)-Rha*p*-(1→	42, 55, 89, 100, 131, 189, 233	1.0026
7.324	1,2,5-O-Ac_3_-3,4,6-O-Me_3_- galactitol	→2)-Gal*p*-(1→	55, 74, 97, 99, 118, 128, 143, 150, 185, 203	0.4839
6.552	1,3,4-O-Ac_3_-2,5-O- Me_2_-arabinitol	→3)-Ara*f*-(1→	43, 57, 71, 87, 101, 117, 129, 145, 168	0.9761

## Data Availability

The original contributions presented in this study are included in the article. Further inquiries can be directed to the corresponding authors.

## References

[1] Meyer A.R., Goldenring J.R. (2018). Injury, Repair, Inflammation and Metaplasia in the Stomach. J. Physiol..

[2] Paulraj R.S., Sathiyaseelan A., Perumal P., Ramachandran A., Paulraj S.G. (2026). A Comprehensive Analysis of Therapeutic Potential of Medicinal Plant Extracts to Treat Ethanol-Induced Gastric Ulcer. Biomedicines.

[3] Tarnawski A.S., Ahluwalia A. (2018). Increased Susceptibility of Aging Gastric Mucosa to Injury and Delayed Healing: Clinical Implications. World J. Gastroenterol..

[4] Cheung K.S., Chan E.W., Wong A.Y.S., Chen L., Wong I.C.K., Leung W.K. (2018). Long-Term Proton Pump Inhibitors and Risk of Gastric Cancer Development after Treatment for *Helicobacter pylori*: A Population-Based Study. Gut.

[5] Zhao Z., Wei G., Wang L., Jiang Y., Zhang X., Fang L., Du G., Kong L. (2024). Pretreatment with Dan-Shen-Yin Granules Alleviates Ethanol-Induced Gastric Mucosal Damage in Rats by Inhibiting Oxidative Stress and Apoptosis via Akt/Nrf2 Signaling Pathway. Phytomedicine.

[6] Yun S.M., Cho J.M., Hong K.S., Lee D.Y., Ji S.D., Son J.G., Kim E.H. (2017). Gastroprotective Effect of Mature Silkworm, *Bombyx mori* against Ethanol-Induced Gastric Mucosal Injuries in Rats. J. Funct. Foods.

[7] Shen C., Zhang S., Di H., Wang S., Wang Y., Guan F. (2025). The Role of Triterpenoids in Gastric Ulcer: Mechanisms and Therapeutic Potentials. Int. J. Mol. Sci..

[8] Xu Y., Lin L., Zheng H., Xu S., Hong X., Cai T., Xu J., Zhang W., Mai Y., Li J. (2024). Protective Effect of *Amauroderma rugosum* Ethanol Extract and Its Primary Bioactive Compound, Ergosterol, against Acute Gastric Ulcers Based on LXR-Mediated Gastric Mucus Secretions. Phytomedicine.

[9] Yan S., Bao S., Chen T., Chen J., Zhang J., Hu X., Liang Y., Zhou X., Li J. (2024). Cinnamaldehyde Alleviates Aspirin-Induced Gastric Mucosal Injury by Regulating Pi3k/Akt Pathway-Mediated Apoptosis, Autophagy and Ferroptosis. Phytomedicine.

[10] Sun C.Y., Li Y.T., Liu D., Chen C.W., Liao M.L. (2025). Gastroprotective Potential of the Aqueous Extract of Nine-Steaming and Nine-Sun-Drying Processed *Polygonatum cyrtonema* Hua against Alcoholic Gastric Injury in Mice. J. Ethnopharmacol..

[11] Zhang Y., Wang H., Mei N., Ma C., Lou Z., Lv W., He G. (2018). Protective Effects of Polysaccharide from *Dendrobium nobile* against Ethanol-Induced Gastric Damage in Rats. Int. J. Biol. Macromol..

[12] Liu H., Zhuang S., Liang C., He J., Brennan C.S., Brennan M.A., Ma L., Xiao G., Chen H., Wan S. (2022). Effects of a Polysaccharide Extract from *Amomum villosum* Lour. on Gastric Mucosal Injury and Its Potential Underlying Mechanism. Carbohydr. Polym..

[13] Lian Y.Z., Lin I.H., Yang Y.C., Chao J.C.J. (2020). Gastroprotective Effect of *Lycium barbarum* Polysaccharides and C-Phycocyanin in Rats with Ethanol-Induced Gastric Ulcer. Int. J. Biol. Macromol..

[14] Wang F., Yuan C., Deng R., Liu Y. (2025). Multi-Omics Analysis Reveals the Pre-Protective Mechanism of *Dendrobium flexicaule* Polysaccharide against Alcohol-Induced Gastric Mucosal Injury. Int. J. Biol. Macromol..

[15] Chen L., Lai Y., Dong L., Kang S., Chen X. (2017). Polysaccharides from *Citrus grandis* L. Osbeck Suppress Inflammation and Relieve Chronic Pharyngitis. Microb. Pathog..

[16] Zhang X., Zheng X., Wang H., Kang C., Jin R., Wang D., Li S., Wang Y., Zhang B., Cao J. (2025). Primary Structural Transformations of Water-Soluble Polysaccharides in the Granulated Juice Sacs of *Citrus changshanensis*. J. Agric. Food Chem..

[17] Li X., Chen J., Yin Y., Xiao S., Zhang R., Yang Y., Li L., Xu H., Zhang X., Hu P. (2024). Chemical Structure Elucidation and Functional Activities Comparison of Two Polysaccharides Purified from *Citrus reticulata* Blanco Peels. Chem. Biol. Technol. Agric..

[18] Zeng S.L., Li S.Z., Lai C.J.S., Wei M.Y., Chen B.Z., Li P., Zheng G.D., Liu E.-H. (2018). Evaluation of Anti-Lipase Activity and Bioactive Flavonoids in the *Citri reticulatae* Pericarpium from Different Harvest Time. Phytomedicine.

[19] Liu X., Huang L., Zhang X., Xu X. (2025). Polysaccharides with Antioxidant Activity: Extraction, Beneficial Roles, Biological Mechanisms, Structure-Function Relationships, and Future Perspectives: A Review. Int. J. Biol. Macromol..

[20] Fazzino F., Mauriello F., Paone E., Sidari R., Calabrò P.S. (2021). Integral Valorization of Orange Peel Waste through Optimized Ensiling: Lactic Acid and Bioethanol Production. Chemosphere.

[21] Liu N., Li X., Zhao P., Zhang X., Qiao O., Huang L., Guo L., Gao W. (2021). A Review of Chemical Constituents and Health-Promoting Effects of Citrus Peels. Food Chem..

[22] Li J.H., Ju G.X., Jiang J., Li N.S., Peng J., Luo X.J. (2016). Lipoic Acid Protects Gastric Mucosa from Ethanol-Induced Injury in Rat through a Mechanism Involving Aldehyde Dehydrogenase 2 Activation. Alcohol.

[23] Liu Y., Li W., Yang L., Tong Z., Wang Z., Zhao Y., Luo D. (2025). Discovery of Ganodecalone a, from Ganoderma Calidophilum for the Treatment of Acute Gastric Ulcers by Binding to ALOX5 via the MAPK/NF-κB/iNOS Signaling. J. Ethnopharmacol..

[24] Lin J., Wang L., Li W., Li Y., Tang F., Xu J., Li W., Gong H., Jiang X., Feng Y. (2024). Dried Tangerine Peel Polysaccharide Accelerates Wound Healing by Recruiting Anti-Inflammatory Macrophages. Int. Immunopharmacol..

[25] Yang W., Huang G. (2023). Preparation and Analysis of Polysaccharide from *Solanum tuberdsm*. Ultrason. Sonochem..

[26] An Q., Ye X., Han Y., Zhao M., Chen S., Liu X., Li X., Zhao Z., Zhang Y., Ouyang K. (2020). Structure Analysis of Polysaccharides Purified from *Cyclocarya paliurus* with DEAE-Cellulose and Its Antioxidant Activity in RAW264.7 Cells. Int. J. Biol. Macromol..

[27] Ren Y., Zhu Z.-Y., Sun H., Chen L.J. (2017). Structural Characterization and Inhibition on α-Glucosidase Activity of Acidic Polysaccharide from *Annona squamosa*. Carbohydr. Polym..

[28] Liang J., Zhao Y., Yang F., Zheng L., Ma Y., Liu Q., Cai L., Gong W., Wang B. (2022). Preparation and Structure-Activity Relationship of Highly Active Black Garlic Polysaccharides. Int. J. Biol. Macromol..

[29] Kim S.Y., Kim E.A., Kim Y.S., Yu S.K., Choi C., Lee J.S., Kim Y.T., Nah J.W., Jeon Y.J. (2016). Protective Effects of Polysaccharides from *Psidium guajava* Leaves against Oxidative Stresses. Int. J. Biol. Macromol..

[30] Liu J., Pu Q., Qiu H., Di D. (2021). Polysaccharides Isolated from *Lycium barbarum* L. by Integrated Tandem Hybrid Membrane Technology Exert Antioxidant Activities in Mitochondria. Ind. Crops Prod..

[31] Yang H., Wang S., Yang L., Song H., Zhang G., He Y., Liu H. (2023). Impact Mechanism of Methylation Degree on Structure and Emulsifying Ability of Soy Hull Polysaccharides. Food Hydrocoll..

[32] Khawas S., Sivová V., Anand N., Bera K., Ray B., Nosáľová G., Ray S. (2018). Chemical Profile of a Polysaccharide from *Psidium guajava* Leaves and It’s In Vivo Antitussive Activity. Int. J. Biol. Macromol..

[33] Meng M., Zhang R., Ning F., Sun Y., Feng Y., He X., Han R., Liu Q., Sun H. (2025). Structural Characterization and Alleviation on Osteoporotic of Polysaccharide Purified from *Psidium guajava* L. Leaves. J. Agric. Food Res..

[34] Li Y., Gao J.N., Liang J., Zhu X.H., Kuang H.X., Xia Y.G. (2025). An Alternative GC–MS Library for Methylation Analysis of Polysaccharides via Partially Methylated Aldononitrile Acetates. Carbohydr. Polym..

[35] Liu X., Ren Z., Yu R., Chen S., Zhang J., Xu Y., Meng Z., Luo Y., Zhang W., Huang Y. (2021). Structural Characterization of Enzymatic Modification of *Hericium erinaceus* Polysaccharide and Its Immune-Enhancement Activity. Int. J. Biol. Macromol..

[36] Lin B., Fan Y., Huang G. (2023). Preparation, Analysis and Properties of Shaddock Ped Polysaccharide and Its Derivatives. Carbohydr. Res..

[37] Yan X., Liu B., Ru G., Feng J. (2021). Preparation and Characterization of Curdlan with Unique Single-Helical Conformation and Its Assembly with Congo Red. Carbohydr. Polym..

[38] Er H., Gemici A., Tas G.G., Sati L., Zengin G., Bilmen S., Derin N., Kelek S.E. (2023). Acetyl-L-Carnitine Attenuates Chronic Ethanol-Induced Oxidative Stress, ER Stress and Apoptosis in Rat Gastric Tissue. Alcohol.

[39] Furevi A., Ruda A., Angles d’Ortoli T., Mobarak H., Ståhle J., Hamark C., Fontana C., Engström O., Apostolica P., Widmalm G. (2022). Complete 1H and 13C NMR Chemical Shift Assignments of Mono-to Tetrasaccharides as Basis for NMR Chemical Shift Predictions of Oligo- and Polysaccharides Using the Computer Program CASPER. Carbohydr. Res..

[40] Wang L., Li W., Li Y., Chen G., Zhao L., Li W., Wang S., Wang C., Feng Y., Zhang Y. (2024). Dried Tangerine Peel Polysaccharide (DTPP) Alleviates Hepatic Steatosis by Suppressing TLR4/MD-2-Mediated Inflammation and Endoplasmic Reticulum Stress. Bioorg. Chem..

[41] Muhidinov Z.K., Nasriddinov A.S., Strahan G.D., Jonmurodov A.S., Bobokalonov J.T., Ashurov A.I., Zumratov A.H., Chau H.K., Hotchkiss A.T., Liu L.S. (2024). Structural Analyses of Apricot Pectin Polysaccharides. Int. J. Biol. Macromol..

[42] Zhou T., Jiang Y., Wen L., Yang B. (2021). Characterization of Polysaccharide Structure in Citrus Reticulate ‘Chachi’ Peel during Storage and Their Bioactivity. Carbohydr. Res..

[43] Chen R., Jin C., Tong Z., Lu J., Tan L., Tian L., Chang Q. (2015). Optimization Extraction, Characterization and Antioxidant Activities of Pectic Polysaccharide from Tangerine Peels. Carbohydr. Polym..

[44] Vityazev F.V., Golovchenko V.V., Patova O.A., Khlopin V.A., Kosolapova N.V., Dmitrenok A.S., Shashkov A.S. (2024). Pectic Polysaccharides of Black Radish Taproots: Extraction, Structural Characterization. Food Chem..

[45] Jin M.Y., Li M.Y., Huang R.M., Wu X.Y., Sun Y.M., Xu Z.L. (2021). Structural Features and Anti-Inflammatory Properties of Pectic Polysaccharides: A Review. Trends Food Sci. Technol..

[46] Vogt L.M., Sahasrabudhe N.M., Ramasamy U., Meyer D., Pullens G., Faas M.M., Venema K., Schols H.A., de Vos P. (2016). The Impact of Lemon Pectin Characteristics on TLR Activation and T84 Intestinal Epithelial Cell Barrier Function. J. Funct. Foods.

[47] Liu Z., Dang J., Wang Q., Yu M., Jiang L., Mei L., Shao Y., Tao Y. (2013). Optimization of Polysaccharides from *Lycium ruthenicum* Fruit Using RSM and Its Anti-Oxidant Activity. Int. J. Biol. Macromol..

[48] Zhang C., Gao F., Gan S., He Y., Chen Z., Liu X., Fu C., Qu Y., Zhang J. (2019). Chemical Characterization and Gastroprotective Effect of an Isolated Polysaccharide Fraction from *Bletilla striata* against Ethanol-Induced Acute Gastric Ulcer. Food Chem. Toxicol..

[49] Cai Y., Liu S., Ge X., Cheng L., Zhang X. (2024). Inhibitory Effect of Tea Flower Polysaccharides on Oxidative Stress and Microglial Oxidative Damage in Aging Mice by Regulating Gut Microbiota. Food Funct..

[50] Xia X., Zhou Q., Su H., Liu L., Chen J., Chen Y., Lin S., Zhou X., Wang J. (2026). Protective Effect of Wild Polysaccharides Extracted from *Ulva prolifera* on Oxidative Stress Damage in Valproic Acid-Induced Neuronal Cells. Front. Pharmacol..

[51] Bermudez-Brito M., Sahasrabudhe N.M., Rösch C., Schols H.A., Faas M.M., De Vos P. (2015). The Impact of Dietary Fibers on Dendritic Cell Responses In Vitro Is Dependent on the Differential Effects of the Fibers on Intestinal Epithelial Cells. Mol. Nutr. Food Res..

[52] Chatterjee A., Khatua S., Chatterjee S., Mukherjee S., Mukherjee A., Paloi S., Acharya K., Bandyopadhyay S.K. (2013). Polysaccharide-Rich Fraction of *Termitomyces eurhizus* Accelerate Healing of Indomethacin Induced Gastric Ulcer in Mice. Glycoconj. J..

[53] Raish M., Ahmad A., Ansari M.A., Alkharfy K.M., Aljenoobi F.I., Jan B.L., Al-Mohizea A.M., Khan A., Ali N. (2018). *Momordica charantia* Polysaccharides Ameliorate Oxidative Stress, Inflammation, and Apoptosis in Ethanol-Induced Gastritis in Mucosa through NF-kB Signaling Pathway Inhibition. Int. J. Biol. Macromol..

[54] Zhang F., Xu H., Yuan Y., Huang H., Wu X., Zhang J., Fu J. (2021). *Lyophyllum decastes* Fruiting Body Polysaccharide Alleviates Acute Liver Injury by Activating the Nrf2 Signaling Pathway. Food Funct..

[55] Lin X., Lan M., Xu C., Pan W., Zhang C., Li F., Xuan W., Chen M., Wang H., Huang M. (2023). Peach Gum Polysaccharides Promotes Epithelial Proliferation to Attenuate Ulcerative Colitis by PI3K/AKT Pathway. J. Funct. Foods.

